# Anti-*Salmonella* Activity and Peptidomic Profiling of Peptide Fractions Produced from Sturgeon Fish Skin Collagen (*Huso huso*) Using Commercial Enzymes

**DOI:** 10.3390/nu13082657

**Published:** 2021-07-30

**Authors:** Maryam Atef, Yasmina Ait Chait, Seyed Mahdi Ojagh, Ali Mohammad Latifi, Mina Esmaeili, Riadh Hammami, Chibuike C. Udenigwe

**Affiliations:** 1 Department of Seafood Science and Technology, Faculty of Fisheries and Environmental Science, Gorgan University of Agricultural Sciences and Natural Resources, Gorgan 4913815739, Iran; atef.m22@gmail.com; 2 School of Nutrition Sciences, Faculty of Health Sciences, University of Ottawa, Ottawa, ON K1H 8M5, Canada; yaitc043@uottawa.ca (Y.A.C.); riadh.hammami@uottawa.ca (R.H.); 3 Applied Biotechnology Research Center, Tehran 19945-546, Iran; amlatifi290@gmail.com; 4 Department of Fisheries, Faculty of Animal Sciences and Fisheries, Sari Agricultural Sciences and Natural Resources University, Sari 48181-68984, Iran; mina.smaily@gmail.com; 5 Department of Chemistry and Biomolecular Sciences, University of Ottawa, Ottawa, ON K1N 6N5, Canada

**Keywords:** sturgeon fish (*Huso huso*), collagen, bioactive peptides, antimicrobial peptides, peptidomics, *Salmonella*

## Abstract

This study investigated peptide fractions from fish skin collagen for antibacterial activity against *Escherichia coli* and *Salmonella* strains. The collagen was hydrolyzed with six commercial proteases, including trypsin, Alcalase, Neutrase, Flavourzyme, pepsin and papain. Hydrolyzed samples obtained with trypsin and Alcalase had the largest number of small peptides (molecular weight <10 kDa), while the hydrolysate produced with papain showed the lowest degree of hydrolysis and highest number of large peptides. Four hydrolysates were found to inhibit the growth of the Gram-negative bacteria, with papain hydrolysate showing the best activity against *E. coli*, and Neutrase and papain hydrolysates showing the best activity against *S. abony*; hydrolysates produced with trypsin and pepsin did not show detectable antibacterial activity. After acetone fractionation of the latter hydrolysates, the peptide fractions demonstrated enhanced dose-dependent inhibition of the growth (colony-forming units) of four *Salmonella* strains, including *S. abony* (NCTC 6017), *S. typhimurium* (ATCC 13311), *S. typhimurium* (ATCC 14028) and *S. chol* (ATCC 10708). Shotgun peptidomics analysis of the acetone fractions of Neutrase and papain hydrolysates resulted in the identification of 71 and 103 peptides, respectively, with chain lengths of 6–22 and 6–24, respectively. This work provided an array of peptide sequences from fish skin collagen for pharmacophore identification, structure–activity relationship studies, and further investigation as food-based antibacterial agents against pathogenic microorganisms.

## 1. Introduction

The increasing prevalence of bacterial resistance to commercial antibiotics leading to failed treatments and increased expenses has become a public health concern [1]. Moreover, the increasing constraint on the use of chemical preservatives and the growing demand for natural antimicrobial agents in foods or food supplements has spurred emerging research towards the discovery of novel natural antimicrobial agents with a broad spectrum of antibiotic activity [2]. Consequently, the development of sustainable alternatives to synthetic antibiotics has become a research priority. Antimicrobial peptides (AMPs) are essential components of the innate immune response and a diverse group of individually unique small peptides (less than 50 amino acid residues) that can participate in different organisms as immune modulators and exhibit broad-spectrum antimicrobial activity towards pathogens (Gram-positive and Gram-negative bacteria, viruses, parasites, protozoa, yeast, and fungi) [3]. Natural AMPs can be isolated from practically every living organism, from prokaryotes to humans. The food source of proteins or peptides with antibacterial activities against Gram-positive and Gram-negative strains is diverse, including whole milk [4], lactoferrin [5], ovotransferrin [6], casein [7] and β-lactoglobulin [8]. Moreover, proteins from fish sources have shown promise as functional food ingredients as they are a valuable source of bioactive peptides. These peptides are encrypted within the primary protein sequence and can be released upon enzymatic hydrolysis [9]. Bioactive peptides isolated from various fish protein hydrolysates have short chain lengths, with only 3–20 amino acid residues, and have shown many bioactivities, such as antithrombotic, immunomodulatory, antihypertensive, anticoagulant, antioxidative, and antimicrobial properties [10,11].

Several studies have reported that treatment of marine organisms by enzymatic hydrolysis yielded AMPs and hydrolysates with activity against pathogenic bacteria. For instance, antibacterial peptide fractions isolated from Atlantic mackerel (*Scomber scombrus*) by-products were reported to inhibit the growth of Gram-positive and Gram-negative bacteria [12]. Sila et al. [13,14] also demonstrated potent inhibitory activity against pathogenic bacteria using antibacterial peptides from barbel muscle protein hydrolysates produced with Alcalase. In addition, antibacterial peptides and fractions have been isolated from pepsin hydrolysate of half-fin anchovy [2] and rainbow trout by-products [15], and from several crab species, including Atlantic rock crab *Cancer irroratus* [16], snow crab *Chionoecetes opilio* [17], blue crab *Callinectes sapidus* [18,19], Chinese mitten crab *Eriocheir sinensis* [20], mud crab *Scylla paramamosain* [21], shore crab *Carcinus meanas* [22], and green crab *Carcinus meanas* [23]. The structural diversity of the marine-derived proteins provides a unique platform for the discovery of a wide range of AMPs for combating several pathogenic bacteria and overcoming antimicrobial resistance.

Fish collagens and their hydrolysates have been used as sources of biologically active peptides with beneficial effects and potential application in the nutraceutical, cosmeceutical, biomedical, and pharmaceutical industries [24]. According to Ennaas et al. [25], collagencin, an antibacterial peptide isolated from fish collagen hydrolysate produced with Protamex, potently inhibited the growth of Gram-positive and Gram-negative bacteria. Considering the need for fish by-product upcycling and the structural diversity of collagen-derived peptides, the objectives of this study were to investigate the antibacterial activities of sturgeon fish skin collagen hydrolysates produced with six different commercial enzymes and to fractionate and identify the collagen peptides with anti-*Salmonella* activity from the most active hydrolysates.

## 2. Materials and Methods

### 2.1. Chemicals

A high-molecular-weight marker was obtained from Bio-Rad Laboratories (Hercules, CA, USA) and N,N,N,N-tetramethyl ethylene diamine (TEMED) was purchased from Sigma Aldrich (St. Louis, MO, USA). Trypsin (from bovine pancreas; ≥7500 U/g) and pepsin (from porcine gastric mucosa; ≥500 U/g) were purchased from Sigma Chemical Co. (St, Louis, MO, USA). Alcalase (protease from *Bacillus licheniformis*; ≥2.4 U/g), Neutrase (protease from *Bacillus amyloliquefaciens*; ≥0.8 U/g), papain (from papaya latex), and Flavourzyme (protease from *Aspergillus oryzae*; ≥500 U/g) were purchased from Sigma Chemical Co. (Bagsveard, Denmark). O-Phthaldialdehyde, 8-anilo-1-naphthalenesulfonic acid (ANS), Coomassie Brilliant Blue R-250, sodium dodecyl sulfate (SDS), and β-mercaptoethanol (βME) were purchased from Merck (Darmstadt, Germany). All other chemicals and reagents used were of analytical grade.

### 2.2. Enzymatic Hydrolysis of Fish Skin Collagen

Collagen was isolated from sturgeon fish skin as previously reported [26]. Lyophilized pepsin-solubilized collagen (1 g) was suspended in 200 mL of deionized water and hydrolyzed separately with six commercial enzymes at their optimal conditions (Table 1) with continuous stirring. During enzymatic hydrolysis, the pH of the mixtures was adjusted by adding 1 M NaOH or 1 M HCl. After 3 h of hydrolysis, the enzymatic reaction was terminated by changing pH as shown in Table 1 to inactivate each of the enzymes. The reaction mixtures were then centrifuged at 10,000× *g* for 30 min at 4 °C and the supernatants lyophilized using a freeze-drier to yield the collagen hydrolysates and stored at −20 °C until use.

### 2.3. Characterization of the Protein Hydrolysates

#### 2.3.1. Degree of Hydrolysis (DH)

DH of the collagen hydrolysates was determined in a 96-well plate using the O-phthaldialdehyde (OPA) method described by Nielsen et al. [27], with some modifications. Briefly, 30 µL sample solution, standard (serine) or blank control (deionized water) was mixed with 225 µL OPA reagent, and after 2 min shaking at room temperature, absorbance the mixtures were measured at 340 nm using a microplate reader. Thereafter, DH was calculated as previously reported [28].

#### 2.3.2. SDS Polyacrylamide Gel Electrophoresis (SDS-PAGE)

The molecular weight profile of the collagen and collagen hydrolysates was determined by SDS-PAGE according to the method of Laemmli [29], with a slight modification, using gradient resolving gel (6%, 9%, 12%, 15%, and 18%) with 3% (*w/v*) stacking gel. To prepare the samples, collagen and collagen hydrolysate powders (1 mg/mL) were dissolved in 0.02 M sodium phosphate buffer (pH 7.2) containing 1% (*w/v*) SDS and 3.5 M urea, and the solutions were gently stirred at 4 °C for 12 h. The mixtures were centrifuged at 5000× *g* for 5 min to remove undissolved debris. Thereafter, the samples were mixed with the sample buffer (2% SDS, 20% glycerol, and 0.5% bromophenol blue in 62.5 mM Tris-HCl buffer, pH 6.8, containing 1 M β-mercaptoethanol), followed by heat denaturation at boiling temperature for 5 min. Electrophoresis was conducted at a constant current at 120 V for 2 h, and the gel was stained overnight with Coomassie Brilliant Blue R-250 solution and distained with Milli-Q water by shaking for 4 h. A standard molecular weight protein marker ranging from 10 to 250 kDa was used to estimate the molecular weight of the collagen peptides. Image scanning of gels was done using the ChemiDoc Imaging System (BioRad Inc., Mississauga, ON, Canada).

#### 2.3.3. Surface Hydrophobicity (*H*_o_)

Surface hydrophobicity (*H*_o_) of the collagen hydrolysates was determined by the fluorescence method using ANS as the hydrophobic probe. Samples at concentrations ranging from 0.005 to 0.025% were mixed with 20 µL ANS in a Grenier UV-Star (96-well) microplate. Fluorescence of the mixture was then measured at excitation and emission wavelengths of 390 and 470 nm, respectively, using a Spark multimode microplate reader (Tecan, Switzerland). The slope of the fluorescence intensity vs. concentration plot was used to represent the surface hydrophobicity (*H*_o_), as previously reported [30]. 

#### 2.3.4. Dynamic Light Scattering (DLS) Analysis

Zeta (ζ)-potential and mean particle size of the collagen hydrolysates were evaluated by DLS using the Zetasizer Nano Series Nano-ZS (Malvern Instruments Ltd., Malvern, UK) at 25 °C. The hydrolyzed samples (0.05 mg/mL) were mixed in water and used to determine the zeta potential and particle size of the samples. All measurements were taken in triplicates at 25 °C with the Smoluchowski model at F (ka) 1.50 and backscattered angle of 173°.

### 2.4. Solvent Fractionation of the Hydrolysates

Selected collagen hydrolysates were further subjected to solvent fractionation to separate the bioactive peptides as previously reported [12]. High-molecular-weight peptides in the samples were precipitated using ice-cold acetone (50%, *v/v*) followed by centrifugation at 8000× *g* for 20 min at 4 °C. Thereafter, acetone in the supernatants containing low-molecular-weight peptides was evaporated using a centrifugal vacuum evaporator, and the samples were freeze-dried to obtain the peptide fraction powders. 

### 2.5. Liquid Chromatography-Tandem Mass Spectrometry (LC-MS/MS) and Peptidomics Data Analysis

Shotgun proteomics was used to identify peptides in the collagen peptide fractions. Lyophilized samples were separated by a 60-min gradient elution at a flow rate of 250 nL/min using an EASY-nLC integrated nano-HPLC system (Thermo Fisher, San Jose, CA, USA), which was directly interfaced with a quadrupole Orbitrap (Q-Exactive) mass spectrometer (Thermo Fisher, San Jose, CA, USA). The analytical column used was a PepMap RSLC EASY-Spray column (75 μm × 50 cm) packed with C18 resin (2 μm). Eluted peptides were then introduced into the Orbitrap Q-Exactive mass spectrometer, operated in the data-dependent acquisition mode using the MaxQuant software with a single full-scan spectrum (400–1500 m/z, 70,000 resolution) followed by 10 data-dependent MS/MS scans in the Orbitrap mass analyzer. For peptide identification, the spectral data were processed with MaxQuant version 1.6.10.43 software (Max Planck Institute of Biochemistry, Planegg, Germany) using the Andromeda peptide search engine, as reported by Tyanova et al. [31].

### 2.6. Antibacterial Activity of Collagen Peptides

Antibacterial activity of the collagen hydrolysates and the acetone fractions was performed using a microtest polystyrene 96-well microplate (VWR Tissue Culture Plates, Randor, PA, USA) as previously described [32]. Briefly, the microplate wells were filled by distributing 100 µL of BHI medium. Medium alone and medium with each strain inoculum were used as blank and control, respectively, on the same microplate. Then, 100 µL of each collagen hydrolysates and acetone fractions prepared in the medium were added to each well (Column 3 to Column 12) and 2-fold diluted with the medium starting from 100 mg/mL for collagen peptides and from 10 mg/mL for acetone fractions. The wells, containing 100 µL of media or sample solution, were inoculated with 100 µL overnight culture of the target strains diluted to a final concentration of 10^5^ CFU/mL. The microplates were subsequently incubated at 37 °C, and absorbance at 650 nm was measured at 20 min intervals for 24 h, using the Spark multimode microplate. Viable bacterial strains were quantified after 6 h incubation with the acetone fractions or BHI medium as control using the standard plate counting method on BHI agar and expressed as CFU/mL. 

### 2.7. Statistical Analysis

Data were collected in triplicate and analyzed using SPSS (Chicago, IL, USA). Results were expressed as mean ± standard deviation, and the differences between mean values of different samples were determined by the Least Significant Difference (LSD) test (*p* < 0.05). 

## 3. Results

### 3.1. Degree of Hydrolysis and Surface Hydrophobicity of the Hydrolysates

**Figure 1:**
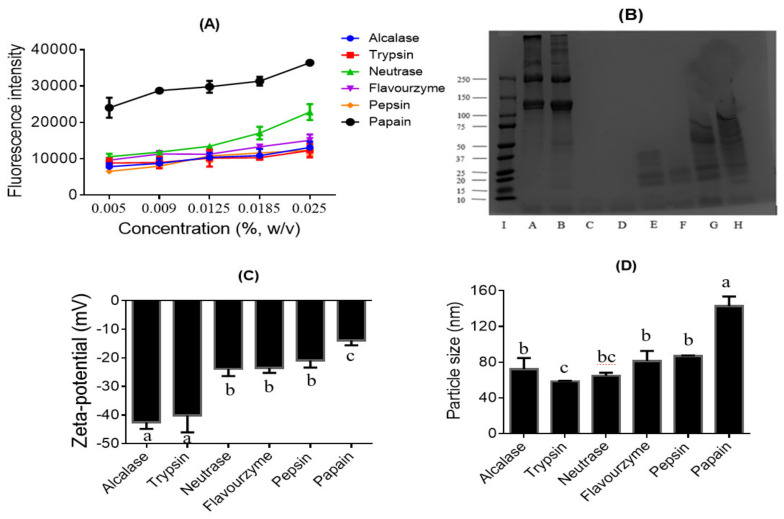
Properties of the collagen protein hydrolysates. (**A**) Surface hydrophobicity (H_0_), (**B**) SDS-PAGE protein profile, (**C**) zeta potential, and (**D**) mean particle size of the collagen hydrolysates produced by Alcalase, trypsin, Neutrase, Flavourzyme, pepsin and papain. Mean values with different letters in (**C**) and (**D**) are significantly different with *p* < 0.05. SDS-PAGE label: Lanes I: High molecular-weight protein marker, A = Acid-solubilized collagen (ASC), B = Pepsin-solubilized collagen (PSC), and collagen hydrolysates obtained using C = trypsin, D = Alcalase, E = Flavourzyme, F = Neutrase, G = pepsin, and H = papain.

DH of the collagen hydrolysates produced with different enzymes (trypsin, Alcalase, Neutrase, Flavourzyme, pepsin, and papain) at their optimal temperature and pH conditions are shown in Table 1. Enzymes were used at the same enzyme–substrate ratio to compare their hydrolytic efficiencies. As shown in Table 1, trypsin and papain hydrolysate demonstrated the highest and lowest DH (*p* < 0.05), respectively, in producing the collagen hydrolysates. Interestingly, there was no significant difference between the DH of hydrolysates obtained with the single enzyme, pepsin and the multi-enzyme, Flavourzme. In contrast, the highest surface hydrophobicity was observed for collagen hydrolysate produced with papain, whereas hydrolysates obtained with Alcalase and trypsin had similar and the lowest *H*_0_ values (Figure 1A). There was no defined pattern in the DH or *H*_0_ with respect to the origin of the enzymes (plant, animal or microbe) used in collagen hydrolysis.

### 3.2. Molecular Weight Distribution of the Collagen Hydrolysates

The molecular weight profiles of the fish skin collagen and its hydrolysates, determined by SDS-PAGE, are shown in Figure 1B. After treating collagen with the six commercial enzymes, the samples obtained with trypsin (lane C) and Alcalase (lane D) displayed peptide bands with the lowest molecular weights (<10 kDa). In contrast, hydrolysates obtained with the pepsin (lane G) and papain (lane H) showed bands with high molecular weights ranging from ~100 to <10 kDa. The molecular weight profile follows a similar pattern as the DH (Table 1); hydrolysates with higher DH showed lower molecular weight bands on the SDS-PAGE gels, indicating extensive protein hydrolysis by the enzymes, and vice versa. Furthermore, SDS-PAGE showed the disappearance of the two major bands present in the original collagen samples (lanes A and B) in all the hydrolysates (lanes C-H), indicating that all six enzymes hydrolyzed the parent protein, although to different extents.

### 3.3. Surface Charge and Particle Size of the Collagen Hydrolysates

**Figure 2:**
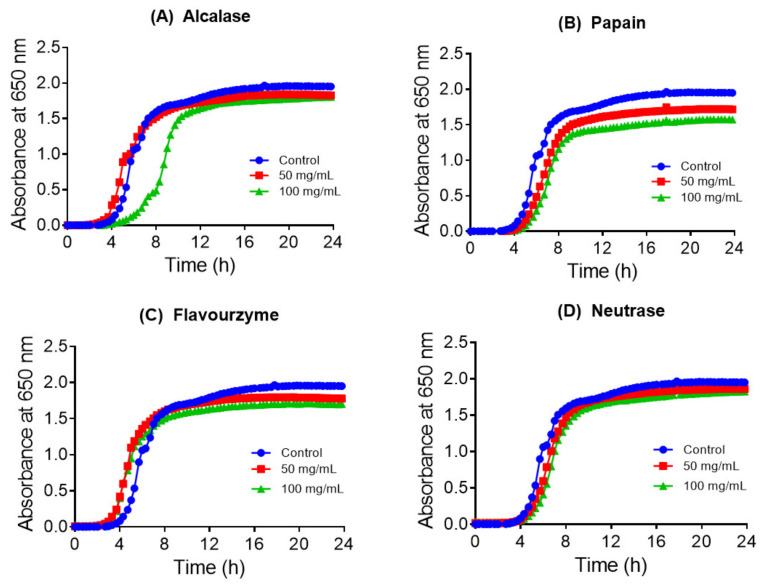
Growth curves of *E. coli* in the absence (control) and presence of collagen hydrolysates produced with four enzymes (**A**) Alcalase, (**B**) papain, (**C**) Flavourzyme, and (**D**) Neutrase. Experiments were conducted three times, and each data point represents the mean value.

As shown in Figure 1C,D, the collagen hydrolysate particles had a net negative surface charge when dispersed in water. The magnitude of zeta potential for the hydrolysates produced with Alcalase and trypsin was significantly higher than the rest, and papain had the lowest surface charge. Conversely, similar particle sizes were observed for the hydrolysates produced with Alcalase, Flavourzyme, and pepsin, while that produce with papain had the highest particle size, and those produced with trypsin and Neutrase showed the lowest values. The particle size results are inversely correlated to the DH (Table 1) and surface charge results (Figure 2C).

### 3.4. Antibacterial Activity of the Collagen Hydrolysates and Peptide Fractions

**Figure 3:**
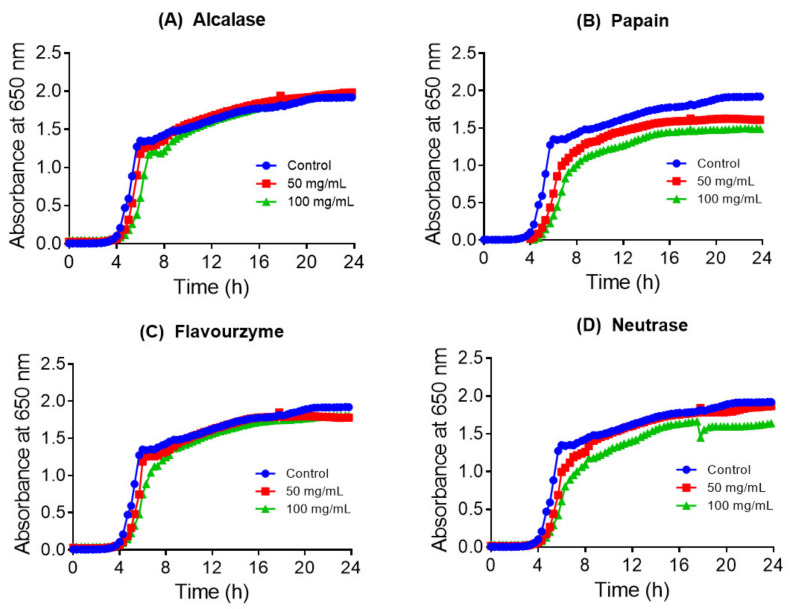
Growth curves of *S. abony* in the absence (control) and presence of collagen hydrolysates produced with four enzymes (**A**) Alcalase, (**B**) papain, (**C**) Flavourzyme, and (**D**) Neutrase. Experiments were conducted three times, and each data point represents the mean value.

The collagen hydrolysates and their acetone fractions were evaluated for inhibitory activity against *E. coli* and four *Salmonella* strains using microdilution assay. As shown in Figure 2 and Figure 3, some collagen hydrolysate at a concentration of 100 mg/mL moderately inhibited the growth of *E. coli* and *S. abony* strains, with crude hydrolysates obtained with papain, Alcalase, and Neutrase being the most potent. Specifically, hydrolysate produced with papain inhibited the growth of both strains, while Neutrase and Alcalase produced hydrolysates with selective antibacterial activity against *E. coli* and *S. abony*, respectively. To a lesser extent, collagen hydrolysate obtained with Flavourzyme exhibited a weak growth inhibitory effect against *S. abony*. Collagen hydrolysates produced with pepsin and trypsin did not show detectable antibacterial activity in both strains under our experimental conditions.

**Figure 4:**
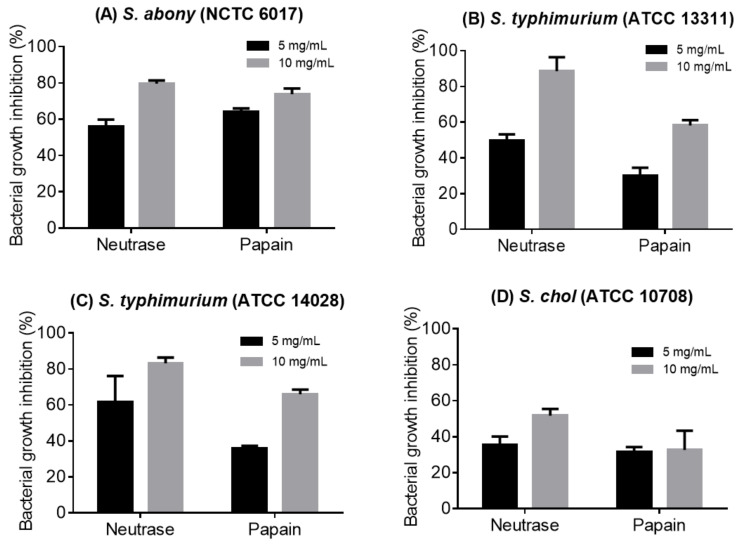
Growth inhibition of *Salmonella* strains in the presence of collagen acetone fractions (**A**): *S. abony* (NCTC 6017); (**B**): *S. typhimurium* (ATCC 13311); (**C**): *S. typhimurium* (ATCC 14028) and (**D**): *S. chol* (ATCC 10708).

Fractionation using acetone increased the specific activity of inhibitory substances in most samples. After 6-h incubation, the acetone fractions of hydrolysates produced with Neutrase and papain exhibited the highest and dose-dependent anti-*Salmonella* growth inhibitory activities (Figure 4). In contrast, fractions from hydrolysates produced with Alcalase and Flavourzyme showed less potency against the four *Salmonella* strains (data not shown). Neutrase produced the hydrolysate with the most potent activity against the four *Salmonella* strains, with the highest growth inhibition of 90% observed at 10 mg/mL against *S. typhimurium* ATCC 13311.

The anti-*Salmonella* activity results were confirmed using the viable bacterial cell counts (CFU) in the presence of acetone peptide fractions of collagen hydrolysates produced with Neutrase and papain (10 mg/mL) after 6 h incubation (Table 2). When treated with Neutrase peptide fraction at 10 mg/mL, the CFU counts of the *Salmonella* strains were significantly (*p* < 0.05) reduced by more than two logs, with *S. typhimurium* (ATCC 13311) being the most sensitive strain (2.28 log reduction). The *Salmonella* strains were less, but significantly, affected by the papain peptide fractions with a log reduction range of 0.32–1.26.

### 3.5. Prole of Peptides in the Acetone Fractions

Peptide profiles of the collagen hydrolysate fractions determined by shogun peptidomics are shown in Table 3 and Table 4 for samples produced with Neutrase and papain, respectively. A total of 71 and 103 peptides were identified in the fractions of collagen hydrolysates produced using Neutrase and papain, respectively, which displayed the most potent antibacterial activities against the Gram-negative bacteria. The length of identified peptides ranged from 6 to 22 and 6 to 24 amino acid residues, and the molecular mass ranged from 612.3 to 2266 Da and 540.2 to 2586 Da for the Neutrase and papain fractions, respectively. The vast majority of peptides in both samples were derived from Type 1 collagen alpha 1 chain, the main collagen protein component. In general, lower molecular weight peptides with 2–5 amino acid residues are less accurately identified by the shotgun peptidomics approach and, if present in the fractions, were not detected in this study.

## 4. Discussion

Biological activities of protein hydrolysates are dependent on the protein substrate, enzyme used for proteolysis, and hydrolysis conditions, such as the enzyme–substrate ratio (E/S), incubation time, temperature, and pH [33]. The DH of the proteins influences the size and structure of the released peptides [34]. Moreover, functional properties of food protein hydrolysates, such as protein solubility, surface hydrophobicity, emulsification, and foaming capacity, depend on the degree of hydrolysis. For instance, the solubility of protein hydrolysate increases with increased DH as observed in samples from yellow stripe trevally [35], Alaska pollack [36], barbel [13,14], anchovy [37], and Atlantic mackerel [12]. High DH would ensure that the peptides become soluble and accessible to interact with their targets in aqueous physiological environments. However, extensive hydrolysis may lead to the loss of the bioactive motif in a peptide. As reported previously, the fish skin collagens in this study consist of two different subunits with an average molecular weight of 110–150 kDa (α1 and α2 at band intensity ratio of 2:1), β (dimers) and small amounts of γ (trimers) [38]. These protein bands were hydrolyzed differently depending on the enzyme used. Interestingly, trypsin gave the highest DH, even higher than multi-enzyme Alcalase and Flavourzyme, resulting in peptides with the lowest MW profiles. Conversely, papain and pepsin gave the lowest DH resulting in several high MW bands. Notably, the original collagen protein bands were not detected in all the samples, signifying their hydrolysis by the enzymes. Similar results have been reported by Suárez-Jiménez et al. [39], Chi et al. [40] and Felician et al. [10], who observed the presence of low molecular weight polypeptide bands for collagens isolated from squid by-products, fish cartilage fish, and jellyfish, respectively.

Surface hydrophobicity of the protein hydrolysates in this study was inversely related to DH. Whereas the smaller sized peptides would be highly soluble, it is expected that the high molecular weight peptides from limited hydrolysis would interact more easily to form hydrophobic pockets or aggregates that bind the fluorescent molecular probe [41,42]. Furthermore, surface charge distribution is an important parameter for the determination of biomolecular structure and interactions in aqueous environments [30]. Results from this study indicated weak electrostatic stabilization (except for hydrolysates produced with Alcalase and trypsin) and inclination of the hydrolysate particles to aggregate in aqueous solution. The net surface charges of the hydrolysates in aqueous solution also decreased in magnitude with an increase in surface hydrophobicity, which is an outcome of peptide aggregation. The molecular profile, surface properties and intermolecular interactions of peptides are important determinants of their bioaccessibility and binding to molecular targets, which would influence their bioactivities.

Antimicrobial peptides have been derived from various food sources by enzymatic hydrolysis, fermentation or gastrointestinal digestion for combating food-borne pathogens [43]. Results in this study revealed that the extent of the antibacterial activity of collagen hydrolysates against the *S. abony* and *E. coli* varies with different enzymes used for hydrolysis. Sila et al. [14] demonstrated that hydrolysates with higher DH (16.2% and 14.53%) or lower DH (2.8%) did not inhibit the growth of Gram-positive and Gram-negative bacteria, compared to hydrolysates with DH 6.6%, which showed antibacterial effect against a broad spectrum of Gram-positive (*Listeria monocytogenes, Staphylococcus aureus, Enterococcus faecalis, Micrococcus luteus and Bacillus cereus*) and Gram-negative bacteria (*Escherichia coli, Salmonella enterica, Pseudomonas aeruginosa, Klebsiella pneumonia, and Enterobacter sp.*). In our study, the DH had no significant effect (*p* > 0.05) on antibacterial activity. [15] and [44] observed similar results in their studies on trout protein hydrolysate and yak kappa-casein hydrolysate, respectively. The biological attributes of peptides are largely influenced by their molecular structural properties, including amino acid composition, sequence, net charge, and chain length [43]. These properties may have influenced the antibacterial activities of the collagen hydrolysates. Notably, hydrophobicity is thought to be important because it facilitates the interaction of the peptides with bacterial cytoplasmic membranes [44]. This feature relates more to the molecular hydrophobicity of individual peptides instead of surface hydrophobicity.

The bioactivity mechanisms of antibacterial peptides can be by interaction with negatively charged cell surface components, such as lipoteichoic acid in the outer membranes of Gram-positive bacteria and lipopolysaccharides in the cell wall of Gram-negative bacteria. Antibacterial peptides produce pores, channels or peptide–lipid complexes in the outer membrane or the cell wall of bacteria, and disruption of cytoplasmic membranes occurs followed by cell lysis, which results in inhibition of cell function or death [43,45]. Because of the increasing bacterial resistance against many antibiotics and advances in peptide design and synthesis, there has been a heightened interest in the discovery of new effective antibiotics [46]. Moreover, food-based sources provide potentially cheaper and safer alternatives to chemical antibacterial agents [43]. In our study, the collagen hydrolysates contained several peptides, some of which may be inactive. Acetone fractionation of the most potent hydrolysates (produced with Neutrase and papain) resulted in significantly increased antibacterial activity against four S. species, even at lower concentrations. This indicates that the downstream processing led to the concentration of active peptides in the resulting fraction. Ennaas et al. [12] reported a similar pattern with acetone fractionation of mackerel by-product hydrolysates with antibacterial activity against Gram-positive (*L. innocua*) and Gram-negative bacteria (*E. coli*). The increase in antibacterial activity after fractionation with acetone was attributed to the hydrophobic nature of the fractionated peptides. Similarly, Ruangsri et al. [47] reported that acetonitrile fractions extracted from different Atlantic cod (*Gadus morhua*) tissues had higher antibacterial activity than the aqueous fractions. 

Peptides present in the acetone fractions are important candidates for further evaluation as antibacterial agents or for identification of important bioactive structural motifs. Several of the fish collagen peptides obtained with Neutrase (71 peptides) and papain (103 peptides) had arginine (R) and/or histidine (H), and/or lysine (K) residues in their sequences. These cationic amino acid residues, in addition to the presence of hydrophobic domains in sequences of antibacterial peptides (AMPs), contribute to the formation of amphiphilic topology for adherence of the peptides to bacterial membrane [43,48]. Furthermore, identified peptides from both samples revealed the high occurrence of glycine (G) and proline (P) residues, which are common structural characteristics of several AMPs [11,12,49,50]. Previously, a 12-mer collagencin (GLPGPLGPAGPK; f291–302 of *Petromyzon marinus* collagen, S4R4C5_PETMA) identified in a fraction from *Scomber scombrus* (Atlantic mackerel) by-product hydrolysate was reported to have antimicrobial activity against six Gram-positive and Gram-negative bacteria, including *L. innocua*, *Lactococcus lactis*, *Carnobacterium divergens*, *Staphylococcus aureus*, *Streptococcus pyogenes*, and *E. coli* [25]. Notably, the C-terminal hexamer fragment of collagencin (GPAGPK) was found in seven peptides in our study, including peptides 13, 14, 18 and 19 released by Neutrase (Table 3) and peptides 19 and 73 released by papain (Table 4). It is likely that these motifs played a role in the antibacterial activity of their respective fractions.

## 5. Conclusions

In this study, fish skin collagen was hydrolyzed with different commercial enzymes to release peptides possessing potent antibacterial activity. The results demonstrated that the degree of hydrolysis had no significant effect on antibacterial activity. Collagen hydrolysate produced with Neutrase and papain showed the most potent inhibitory activity against Gram-negative bacteria (*Salmonella* strains), and viable count confirmed the decrease of the cell population. Acetone fractionation significantly enhanced the growth inhibitory activity against four *Salmonella* strains, providing products with stronger potential for use against food-borne pathogens. Antibacterial peptides not only alter the cytoplasmic membrane but also inhibit intracellular targets such as nucleic acid synthesis, protein synthesis or enzymatic activity. Therefore, the large number of peptide sequences identified in the fractions by shotgun peptidomics suggest further investigation of a potentially multi-targeted approach to the antimicrobial effects. Taken together, this study provided an array of peptide sequences from the fish skin by-products for elucidating molecular mechanisms and further exploration as value-added antimicrobial nutraceutical products against food-borne pathogens.

## Figures and Tables

**Table 1:** Hydrolysis conditions and degree of hydrolysis of Sturgeon fish skin collagen with various commercial proteases.

Enzyme	Optimum Conditions					DH (%)
	E/S	Time (h)	Temp. (°C)	pH	pH (Inactivation)	
Trypsin	1:100	3	37	8	3	40.35 ± 0.07 ^a^
Alcalase	1:100	3	50	8	4	24.37 ± 0.01 ^b^
Neutrase	1:100	3	50	8	4	19.63 ± 0.01 ^c^
Flavourzyme	1:100	3	50	7	4	14.43 ± 0.03 ^d^
Pepsin	1:100	3	37	2	6.5–8	12.35 ± 0.02 ^d^
Papain	1:100	3	37	6.5	3	7.38 ± 0.01 ^e^

E/S, enzyme–substrate ratio; DH, degree of hydrolysis. Mean values in the DH column with different letters are significantly different with *p* < 0.05.

**Table 2:** Cell viable counts (log CFU/mL) of *Salmonella* strains in the presence of collagen acetone peptide fractions derived from two enzymatic hydrolysates.

Strains	Control	Neutrase	Papain
*S. abony* (NCTC 6017)	9.30 ± 0.22 ^a^	7.83 ± 0.12 ^b^	8.67 ± 0.10 ^a^
*S. chol* (ATCC 10708)	9.10 ± 0.17 ^a^	8.54 ± 0.10 ^a^	8.78 ± 0.21 ^a^
*S. typhimurium* (ATCC 13311)	9.15 ± 0.11 ^a^	6.87 ± 0.21 ^b^	7.89 ± 0.12 ^b^
*S. typhimurium* (ATCC 14028)	9.21 ± 0.12 ^a^	7.56 ± 0.12 ^b^	7.81 ± 0.12 ^b^

Control, bacteria in BHI medium without the peptide treatment; Neutrase and Papain, bacterial treated with 10 mg/mL of acetone fraction of collagen hydrolysates produced with Neutrase and papain, respectively. Different letters in each row represent significantly different mean values (*p* < 0.05).

**Table 3:** Collagen peptides identified in the acetone fraction of the hydrolysate produced with Neutrase.

No	Sequence	Chain Length	Mass (Da)	Net Charge	Fragment Position	Protein Name
1	AAGDAGKPGERG	12	1084.5261	−2;3	581–592	Type1 collagen alpha1 chain
2	AAGPPGATGFPG	12	998.48214	−2	860–871	Type1 collagen alpha1 chain
3	AAGPPGATGFPGAAGR	16	1353.6789	−2	860–875	Type1 collagen alpha1 chain
4	AEIKAQY	7	821.42832	−2	274–280	
5	AGEELWSLLAD	11	1202.5819	+2	562–572	Afamin
6	AGPPGADGQAGAK	13	1095.5309	−2	807–819	Type1 collagen alpha1 chain
7	ASGPAGPRGPA	11	936.47773	−2	1127–1137	Type1 collagen alpha1 chain
8	ASGPAGPRGPAGPA	14	1161.5891	−2	1127–1140	Type1 collagen alpha1 chain
9	ATGPAGARGSPGSPGND	17	1467.6702	−2	683–699	Type1 collagen alpha1 chain
10	CHRWVSL	7	956.46506	+2	428–434	
11	DAFLGSFLYEY	11	1323.6023	+2	347–357	Serum albumin
12	DFGFVAQ	7	782.3599	−1	1190–1196	Type1 collagen alpha1 chain
13	DGAKGDSGPAGPK	13	1155.552	−3	267–279	Type1 collagen alpha1 chain
14	DGAKGDSGPAGPKGEPGSSGE	21	1855.8184	−2;3	267–287	Type1 collagen alpha1 chain
15	DQLEGALQQ	9	1000.4825	+2	399–407	Keratin, type II cytoskeletal 72
16	DVNRDDACDLLV	12	1403.635	+2	256–267	Inter-alpha-trypsin inhibitor heavy…
17	ENEVALRQSVE	11	1272.631	−2	239–249	Keratin, type I cytoskeletal 10
18	FSGLDGAKGDSGPAGPK	17	1559.758	+3	263–279	Type1 collagen alpha1 chain
19	FSGLPGPTGEPGKQGPGGPSGE	22	2008.949	−3	965–986	Type1 collagen alpha1 chain
20	FYAPELLYYANK	12	1490.7446	+2	172–183	Serum albumin
21	GAAGDAGKPGE	11	928.42502	+2	580–590	Type1 collagen alpha1 chain
22	GAAGDAGKPGERG	13	1141.5476	+2;3	580–592	Type1 collagen alpha1 chain
23	GERGFPGERGGPG	13	1271.6007	+3	670–682	Type1 collagen alpha1 chain
24	GFPGERGGPGA	11	1000.4726	−2	673–683	Type1 collagen alpha1 chain
25	GGDGAPGKDGIRG	13	1155.5632	−3	745–757	Type1 collagen alpha1 chain
26	GGDGAPGKDGIRGM	14	1286.6037	+3	745–758	Type1 collagen alpha1 chain
27	GGPGAKGEVGPAGGRGSDGPQGARG	25	2148.042	−3	340–364	Type1 collagen alpha1 chain
28	GGPGATGPAGAR	12	967.48354	+2	679–690	Type1 collagen alpha1 chain
29	GKNGDRGESGPAGPA	15	1368.6382	−2	1054–1068	Type1 collagen alpha1 chain
30	GKNGDRGESGPAGPAGPA	18	1593.7495	−2	1054–1071	Type1 collagen alpha1 chain
31	GKNGDRGESGPAGPAGPAGPA	21	1818.8609	−2	1054–1074	Type1 collagen alpha1 chain
32	GKNGDRGESGPAGPAGPAGPAGA	23	1946.9195	−2	1054–1076	Type1 collagen alpha1 chain
33	GKNGDRGESGPAGPAGPAGPAGAR	24	2103.0206	−3	1054–1077	Type1 collagen alpha1 chain
34	GKNGDRGESGPAGPAGPAGPAGARG	25	2160.042	−2;3	1054–1078	Type1 collagen alpha1 chain
35	GPAGPAGARG	10	809.4144	+2	1069–1078	Type1 collagen alpha1 chain
36	GPAGPRGPA	9	778.40859	+2	1129–1137	Type1 collagen alpha1 chain
37	GPAGPRGPAGPA	12	1003.5199	−2	1129–1140	Type1 collagen alpha1 chain
38	GPSGPQGAR	9	825.40931	−2	238–246	Type1 collagen alpha1 chain
39	GSRGSPGERGESGPPGPAG	19	1707.7925	−2	787–805	Type1 collagen alpha1 chain
40	IGPAGPPGTPGPPGPPGPPGGGFD	24	2048.9956	+2	1167–1190	Type1 collagen alpha1 chain
41	IVGLPGQRGERG	12	1237.6891	+2	953–964	Type1 collagen alpha1 chain
42	KPKYGLVTY	9	1067.6015	+2	305–313	
43	LDGAKGDSGPAGPK	14	1268.6361	−2;3	266–279	Type1 collagen alpha1 chain
44	LGRVVDP	7	754.43374	+2	443–449	
45	LQAETEGL	8	859.42871	−2	333–340	
46	LQMDYSK	7	883.41095	+2	255–261	Thyroxine-binding globulin
47	LSGAPGEAGREG	12	1099.5258	+2	998–1009	Type1 collagen alpha1 chain
48	LTGSPGSPGPDGKTGPAGPAGQ	22	1904.9228	+2	533–554	Type1 collagen alpha1 chain
49	LTYTSNDSALFILPDKGKM	19	2113.0765	+2	259–277	Serpin A3–4
50	MESTEVFTKKT	11	1299.6381	+2	141–151	
51	MNRDSNKNTLI	11	1304.6507	+2	1434–1444	
52	NGDRGESGPAGPAGPAGPAGAR	22	1917.9041	−3	1056–1077	Type1 collagen alpha1 chain
53	PGAAGPA	7	539.27036	−1	839–845	
54	QDPVTGLTVN	10	1042.5295	+2	681–690	Inter-alpha-trypsin inhibitor heavy…
55	QLQISVDQHGDNLKNTKSEI	20	2266.1553	+2	410–429	Cytokeratin-4
56	RADLERQ	7	886.46208	+2	377–383	Keratin, type I cuticular Ha8
57	RGDKGEAGEAGERG	14	1387.644	−2;3	1086–1099	Type1 collagen alpha1 chain
58	RGESGPAGAPGAPGAPGA	18	1475.7117	−2	1029–1046	Type1 collagen alpha1 chain
59	RGESGPPGPAGF	12	1127.536	−2	795–806	Type1 collagen alpha1 chain
60	RGPPGPMGPPG	11	1018.5018	−2	987–997	Type1 collagen alpha1 chain
61	RGPPGPMGPPGL	12	1131.5859	+2	987–998	Type1 collagen alpha1 chain
62	SAGAQGARGDKGEAGE	16	1459.6651	+2	1079–1094	Type1 collagen alpha1 chain
63	SAGAQGARGDKGEAGEAGER	20	1872.8674	+3	1079–1098	Type1 collagen alpha1 chain
64	SGAPGEAGREG	11	986.44174	+2	999–1009	Type1 collagen alpha1 chain
65	SGAPGEAGREGAAG	14	1185.5374	+2	999–1012	Type1 collagen alpha1 chain
66	SRTSFSSVSRS	11	1199.5895	+2	28–38	
67	TSGLLGAHASAITA	14	1268.6725	+2	1182–1195	
68	VAGAPGALG	9	711.39154	−2	593–601	Type1 collagen alpha1 chain
69	VGATGPKGSRG	11	985.53049	+2	849–859	Type1 collagen alpha1 chain
70	VPGQRG	6	612.33436	−1	424–429	
71	VRLCPG	6	700.36903	−2	347–352	Alpha-2 HS-glycoprotein

**Table 4:** Collagen peptides identified in the acetone fraction of the hydrolysate produced with papain.

No	Sequence	Chain Length	Mass (Da)	Net Charge	Fragment Position	Protein Name
1	AAGDAGKPGERG	12	1084.5261	−2;3	581–592	Type1 collagen alpha1 chain
2	AGPAGPAGAR	10	823.43005	−2	1068–1077	Type1 collagen alpha1 chain
3	AGPPGADGQAGA	12	967.43592	−2	807–818	Type1 collagen alpha1 chain
4	AGPPGADGQAGAKGEPGDS	19	1637.7281	−2	807–825	Type1 collagen alpha1 chain
5	AGRPGEPGPAGPPGPTGE	18	1599.7641	−2;3	909–926	Type1 collagen alpha1 chain
6	AKGEPGDSGAKGDAG	15	1315.6004	−2;3	818–832	Type1 collagen alpha1 chain
7	AKGETGPAGAPG	12	1011.4985	+2	701–712	Type1 collagen alpha1 chain
8	AKIQLCPPPPQVPNACDMTTTV	22	2437.1804	+2	806–827	Complement factor H
9	APDPFRHY	8	1001.4719	−2	1202–1209	Type1 collagen alpha1 chain
10	APGEAGREGAAG	12	1041.4839	+2	1001–1012	Type1 collagen alpha1 chain
11	APGEKGESGPAGPGGPTG	18	1521.7059	−2	770–787	Type1 collagen alpha1 chain
12	APGEKGESGPAGPGGPTGS	19	1608.738	−2	770–788	Type1 collagen alpha1 chain
13	APGFPGGPGA	10	826.39735	+2	335–344	Type1 collagen alpha1 chain
14	ARGSPGSPGNDGAKGETGPAG	21	1838.8507	−2	689–709	Type1 collagen alpha1 chain
15	ASGPAGPRGPAGPAGSSGKD	20	1692.818	−2;3	1127–1146	Type1 collagen alpha1 chain
16	ASGPAGPRGPAGPAGSSGKDGVSG	24	1992.9613	−2;3	1127–1150	Type1 collagen alpha1 chain
17	ATEAGHSAAAWLLTAQGSGTHSPL	24	2333.14	+3	53–76	Peptidoglycan recognition protein 2
18	DEGQDDRPKVGLG	13	1384.6583	+2	34–46	Fibrinogen beta chain
19	DGAKGDSGPAGPKGEPGSSGE	21	1855.8184	−2;3	267–287	Type1 collagen alpha1 chain
20	DGHARGDSVSQGTGLAPGSP	20	1864.8664	+3	270–289	Fibrinogen alpha chain
21	DKGRLQSELKTMQD	14	1647.825	+3	279–292	Cytokeratin-4
22	DSALQLQDFYQEVANPLMTSVAF	23	2586.2312	+3	446–468	Inter-alpha-trypsin inhibitor heavy...
23	DSGGPLACEKNG	12	1203.519	+2	576–587	
24	EKGESGPAGPGGPT	14	1239.5731	+2	773–786	Type1 collagen alpha1 chain
25	EKGEYFAFLETYGT	14	1653.7563	+2	336–349	Complement component C9
26	EKIGCSQPPQIDHG	14	1564.7304	+2	866–879	Complement factor H
27	ENGLQQLTFPLSSE	14	1561.7624	+2	184–197	Alpha-2-macroglobulin
28	ERGFPGE	7	790.36097	−2	671–677	Type1 collagen alpha1 chain
29	EVVSLTVTCCAE	12	1366.6109	+2	66–77	Vitamin D binding protein
30	EWNASQVLANLTW	13	1530.7467	+3	308–320	Alpha-2-antiplasmin
31	FMQSVTGWNMGRAL	14	1596.7541	+2	245–258	Angiotensinogen
32	GAAGDAGKPGERGVA	15	1311.6531	+3	580–594	Type1 collagen alpha1 chain
33	GAAGPKGGPGE	11	896.43519	+2	493–503	Type1 collagen alpha1 chain
34	GADGQAGAKGEPG	13	1113.5051	+2	811–823	Type1 collagen alpha1 chain
35	GAKGDAGSPGPAGPTG	16	1295.6106	+2	826–841	Type1 collagen alpha1 chain
36	GARGDKGEAGEAGE	14	1302.58	−2;3	1084–1097	Type1 collagen alpha1 chain
37	GDRGESGPAG	10	901.38897	+2	1027–1036	Type1 collagen alpha1 chain
38	GEPGDSGAKGDAGSPGPAGPTG	22	1837.8078	−2	820–841	Type1 collagen alpha1 chain
39	GEPGPGGVQ	9	796.37153	−2	442–450	Type1 collagen alpha1 chain
40	GEVGPAGGRGSDGPQGA	17	1467.6702	+2	346–362	Type1 collagen alpha1 chain
41	GFPGADGAAGPKG	13	1100.5251	+2	487–499	Type1 collagen alpha1 chain
42	GFPGPKGAAGDAGKP	15	1325.6728	+2	574–588	Type1 collagen alpha1 chain
43	GGDGAPGKDGIR	12	1098.5418	+2	745–756	Type1 collagen alpha1 chain
44	GGDGAPGKDGIRGM	14	1286.6037	+3	745–758	Type1 collagen alpha1 chain
45	GGPGATGPAGA	11	811.38243	+2	679–689	Type1 collagen alpha1 chain
46	GHRGFTGL	8	843.43514	+2	1102–1109	Type1 collagen alpha1 chain
47	GIAGQRGIVG	10	926.52976	−2	946–955	Type1 collagen alpha1 chain
48	GLVGPKGDTGE	11	1028.5138	+2	67–77	Adiponectin
49	GMKGCPAVMPIDHVYGTLGI	20	2115.0315	+2	88–107	periostin isoform X7
50	GPAGPAGPAG	10	750.36605	−2	1063–1072	Type1 collagen alpha1 chain
51	GPAGPAGSSGK	11	884.43519	+2	1135–1145	Type1 collagen alpha1 chain
52	GPAGPRGPA	9	778.40859	−2	1129–1137	Type1 collagen alpha1 chain
53	GPAGPRGPAGPAG	13	1060.5414	+2	1129–1141	Type1 collagen alpha1 chain
54	GPMGPRGPPGPA	12	1089.5389	−2	175–186	Type1 collagen alpha1 chain
55	GPMGPRGPPGPAG	13	1146.5604	−2	175–187	Type1 collagen alpha1 chain
56	GPRGPPGPAG	10	861.4457	+1	178–187	Type1 collagen alpha1 chain
57	GRSGRSGSFLYQ	12	1313.6476	+2	2406–2417	Truncated profilaggrin
58	GRSRSFLYQVSSHE	14	1651.8067	+3	1436–1449	Truncated profilaggrin
59	GSAGAQGARGDKGEAGE	17	1516.6866	−2	1078–1094	Type1 collagen alpha1 chain
60	GSIQIENGYFVHYF	14	1672.7886	+3	251–264	Inter-alpha-trypsin inhibitor heavy...
61	GSPGERGESGPPGPAG	16	1407.6379	+2	790–805	Type1 collagen alpha1 chain
62	GSPGSPGNDGAKGETGPAG	19	1611.7125	−2	691–709	Type1 collagen alpha1 chain
63	GVCISSLSCSRVGS	14	1467.681	+2	46–59	
64	HRGFSGL	7	772.39802	+2	260–266	Type1 collagen alpha1 chain
65	HRGFTGL	7	786.41367	−2	1103–1109	Type1 collagen alpha1 chain
66	IRDVWGIEGPID	12	1368.7038	+2	192–203	vitronectin
67	KGDAGSPGPAGPTG	14	1167.552	+2	828–841	Type1 collagen alpha1 chain
68	KNGDRGESGPAGPAGPAGPA	20	1761.8394	−2	1055–1074	Type1 collagen alpha1 chain
69	KNGDRGESGPAGPAGPAGPAG	21	1818.8609	−2	1055–1075	Type1 collagen alpha1 chain
70	KNGDRGESGPAGPAGPAGPAGA	22	1889.898	−2	1055–1076	Type1 collagen alpha1 chain
71	KNGDRGESGPAGPAGPAGPAGAR	23	2045.9991	−3	1055–1077	Type1 collagen alpha1 chain
72	KSENARLVLQI	11	1269.7405	+3	158–168	Keratin, type I cuticular Ha6
73	LDGAKGDSGPAGPK	14	1268.6361	+3	266–279	Type1 collagen alpha1 chain
74	LGIANPATDF	10	1017.5131	−2	728–737	Inter-alpha-trypsin inhibitor heavy...
75	LMGEVARHSVQDGK	14	1525.7671	+3	103–116	Peptidoglycan recognition protein 2
76	LPGPTG	6	540.29076	−1	968–973	
77	LPGPTGEPGKQGPGGPSGE	19	1717.8271	+2	968–986	Type1 collagen alpha1 chain
78	LVDTELNCTVLQMD	14	1649.7641	+2	245–258	Thyroxine-binding globulin
79	MHGLISDAEERGER	14	1598.7471	+2	409–422	Keratin, type II cytoskeletal 1b
80	MSAPGPMGPMGPRGPPGPAG	20	1817.8375	+3	168–187	Type1 collagen alpha1 chain
81	MSAPGPMGPMGPRGPPGPAGSN	22	2018.9125	+2	168–189	Type1 collagen alpha1 chain
82	NGDRGESGPAGPAGPAGPAGA	21	1761.803	−2	1056–1076	Type1 collagen alpha1 chain
83	NGDRGESGPAGPAGPAGPAGAR	22	1917.9041	−2;3	1056–1077	Type1 collagen alpha1 chain
84	PAGPAGQDGRAGPPGPSGARG	21	1828.8929	+3	548–568	Type1 collagen alpha1 chain
85	PGPTGEPGKQGPGGPSGE	18	1604.7431	−2	969–986	Type1 collagen alpha1 chain
86	PYRVYCDMKTEKG	13	1645.7592	+2	269–281	Fibrinogen beta chain
87	QLEPEE	6	743.33375	+1	234–239	Complement C3
88	RGDKGEAGEAGE	12	1174.5214	−2	1086–1097	Type1 collagen alpha1 chain
89	RGEGGPAGAPGF	12	1071.5098	+2	624–635	Type1 collagen alpha1 chain
90	RGESGPAGPAGPAGPAGA	18	1475.7117	+2	1059–1076	Type1 collagen alpha1 chain
91	RGPPGPMGPPG	11	1018.5018	+2	987–997	Type1 collagen alpha1 chain
92	RGSAGAQGARGDKGEAGE	18	1672.7877	+3	1077–1094	Type1 collagen alpha1 chain
93	RGSAGAQGARGDKGEAGEA	19	1743.8248	−3	1077–1095	Type1 collagen alpha1 chain
94	RGSPGSPGNDGAKGETGPAG	20	1767.8136	−2	690–709	Type1 collagen alpha1 chain
95	SGAPGEAGREGAAGN	15	1299.5804	−2	999–1013	Type1 collagen alpha1 chain
96	SGAPGEAGREGAAGNEGAPGRD	22	1981.8838	+2	999–1020	Type1 collagen alpha1 chain
97	SGPAGPRGPAGPA	13	1090.552	+2	1128–1140	Type1 collagen alpha1 chain
98	SGPPGPAG	8	638.30239	+1	798–805	Type1 collagen alpha1 chain
99	SRGERGFPGERGGPGATGPAG	21	1968.9514	+3	668–688	Type1 collagen alpha1 chain
100	SVMADATSVPVTE	13	1305.6122	+2	25–37	Protein HP-25 homolog 2
101	VAQPSQE	7	757.36063	−2	1194–1200	Type1 collagen alpha1 chain
102	VKGGDGAPGKDGIRG	15	1382.7266	−2	743–757	Type1 collagen alpha1 chain
103	VKGGDGAPGKDGIRGM	16	1513.7671	+3	743–758	Type1 collagen alpha1 chain

## Data Availability

Data supporting the findings are available within the article.
